# Expanding the scope and relevance of health interventions: Moving beyond clinical trials and behavior change models

**DOI:** 10.3402/qhw.v9.24743

**Published:** 2014-07-21

**Authors:** Khary K. Rigg, Hilary H. Cook, John W. Murphy

**Affiliations:** 1 Department of Mental Health Law & Policy, Louis de la Parte Florida Mental Health Institute, University of South Florida, Tampa, FL, USA; 2 Social Sciences Department, Miami-Dade College, Miami, FL, USA; 3 Department of Sociology, University of Miami, Coral Gables, FL, USA

**Keywords:** health interventions, clinical trials, behavior change models, photovoice, ethnography, narratives, theory, public health

## Abstract

An overemphasis on clinical trials and behavior change models has narrowed the knowledge base that can be used to design interventions. The overarching point is that the process of overanalyzing variables is impeding the process of gaining insight into the everyday experiences that shape how people define health and seek treatment. This claim is especially important to health decision-making and behavior change because subtle interpretations often influence the decisions that people make. This manuscript provides a critique of traditional approaches to developing health interventions, and theoretically justifies what and why changes are warranted. The limited scope of these models is also discussed, and an argument is made to adopt a strategy that includes the perceptions of people as necessary for understanding health and health-related decision-making. Three practical strategies are suggested to be used with the more standard approaches to assessing the effectiveness and relevance of health interventions.

Epidemiology is the study of population health. This work is most commonly done by empirical assessments of populations, in order to identify risk factors and calculate the odds that persons with certain characteristics will contract a disease. Epidemiologists and public health practitioners use the concept of risk to link various sociodemographic factors to the incidence or prevalence of a problem. Risk factors are used, in part, because they provide fast and simple answers to guide public health policy.

This strategy has been referred to as “black box” epidemiology (Susser & Susser, 1996). What is suggested by this phrase is that very little insight is gained into how persons view or will likely respond to certain social or environmental conditions by the use of this model. The so-called objective features of a community may indicate that undesirable conditions are present (e.g., poverty, violence), but how persons interpret these factors is equally important for dealing with health and illness. For this reason, Krieger (2005) recommends that epidemiologists should focus on data that are more “embodied” in communities.

However, epidemiologists and others who work in the area of public health (e.g., social epidemiologists, community psychologists) have tried to be holistic and attuned to community sentiment. For example, one of the major organizing concepts within modern public health is the epidemiological triad (Page, Cole, & Timmreck, 1995). This triad includes an agent, host, and environment as important considerations. This conceptualization of what epidemiology encompasses demonstrates a high level of flexibility and holism, but most studies are often relatively shallow. And although this strategy may include contextual variables, how these factors are interpreted is not necessarily the focus of attention. Most often, several convenient contextual variables are highlighted but merely treated as supplementary factors.

Especially noteworthy is that public health is still heavily burdened by the influence of a biomedical model because of the historical context of the discipline. At its inception as a discipline in the 19th century, epidemiology sought to address the spread of infectious diseases. As part of this legacy, today's standard epidemiological methods are not focused on theoretical premises, but on improving methodological and statistical designs (Morabia, 2007; Saracci, 2007). Generally, the emphasis of public health research is on refining clinical trial approximations through the use of increasingly complex mathematical models of health problems. But critics claim that this methodology is inappropriate for a field that is supposed to be as much about humans in their environmental context as pathogens and disease (Agar, 2003; Krieger, 2012).

Because quantitative indicators seem straightforward and manageable as a starting point, policy makers often rely on such studies to inform interventions. Although this strategy can be useful, especially for rapid assessment and emergency response, these studies may not capture the reality of the persons who will be affected by such programs. As a result, many development projects have the potential to be irrelevant and, in some cases, harmful. Other writers (Biehl, 2007) have shared similar concerns about the dehumanizing potential that health interventions can have when persons’ everyday experiences and perspectives are not seriously considered.

## Advances in public health research

Like all disciplines, public health has gone through different stages of development (Tulchinsky & Varivakova, 2009). The first era of public health was characterized by the sanitation revolution and general environmental and hygienic advances. The second stage focused on individual health behaviors, and is the target of the critique undertaken. The third era of public health is what has been called for since the 1970s, but it is yet to be fully realized. The third era is described by the World Health Organization's “Health for All” goals, and abandons the emphasis on individual level behaviors in exchange for understanding health more comprehensively and aiming to improve quality of life as a human right (Kickbusch, 2003).

One of the major institutional documents that changed the way that public health is approached originated in Canada in 1974 and is called the Lalonde Report (Lalonde, 1981). This document set off what is considered the third era of public health and challenged the purely biomedical view of health. This document was the beginning of a redefinition of health promotion that shifts the focus from individual interventions and supports a holistic approach to health.

In the mid-1980s, an international discussion began that embraces this new public health. Essentially, several important documents were published by the World Health Organization that situated health outcomes at the center of development policy, redefined the goal of health policies to include social and economic well-being, and called for a serious reorientation away from individual risk factors toward addressing context and meaning in the social environment (Kickbusch, 2003). Simultaneous discussion in academia stressed the need to modernize the field of public health that led to an attempt to promote a model that better connects the needs of people and communities, along with broadening of the disciplinary base to include other fields that address more ecologically the health of populations. Although much research in the discipline remains stuck in the second wave, the individual and behaviorally focused era of public health, there are some writers that have accepted the challenge to move public health forward. Agar (2003), for example, outlines how the epidemiological triad of host-agent-environment paradigm that runs through epidemiology can be greatly enhanced by broadening this scheme to consider the “person-in-context.”

## Traditional epidemiology and epistemology

A major philosophical influence at the time epidemiology emerged, and still operative today in most sciences, was René Descartes (1985) who argued that the way to truth is to separate the subjective (mind) from the objective (body). This Cartesian dualism is the epistemology that sustains the scientific method and led to the use of the experimental design as the gold standard for clinical medicine (Zaner, 1988). As a consequence of this dualism, the study of human illness became viewed as a natural (objective) science, whereby subjectivity (human experience) was reduced in importance (Winkelman, 2009). Accordingly, the biomedical model began to permeate the field of epidemiology. The success of the biomedical model is based on imagery that was heavily influenced both by Louis Pasteur's work in germ theory in the mid-1800s and bacteriology that gained momentum with Robert Koch's breakthroughs in the later part of that century. Nonetheless, dualism lent credibility to this emergent type of causal analysis.

During Koch's time of great scientific advancements, physicians viewed bacteriology as the way of improving health and believed that focusing on bacteria was the correct path to cure disease (Gradmann, 2009). This belief is still dominant today, and bacteriology's reductionism and isolationism permeates both medical practice and the field of epidemiology, in the form of searching for unique causal agents.

Hence, epidemiology and medicine has the methodological goal of implementing experimental designs, but mostly through the use of sophisticated statistical models. The question remains whether this method is the best way to understand the intersection of pathogens and the human context. In other words, researchers are supposed to study disease in the human context, and not merely the biological transmission of pathogens. In this regard, experiments create a very strict situation with minimal variety. This process severely limits what is considered relevant to studies in health. Nonetheless, the assumption is that general laws can be found through these restricted cases, and moreover assumes that these laws are similar to those found in physics (Cassell, 1991; Reiser, 1981). This elevation of experimental design, especially in relation to something as tentative as human decision-making relating to health, may be too reductionist to generate meaningful insights.

This traditional outlook assumes that people inhabit a “real world” made of objects with properties that exist outside of interpretation (Murphy & Min Choi, 1997). Variables such as socioeconomic status or poor mental health are assumed to impinge on individual behavior in predictable ways. This viewpoint supports today's dominant perspective on society and generally reinforces the logic and use of biomedicine to study disease (Engel, 1977). Nonetheless, the social and behavioral sciences may not be acquiring knowledge that is relevant to actual human beings. In the health field in particular, applying the “gold standard” randomized control trial, without including other approaches, decouples the medical from the human and may result in complete irrelevance to everyday life and the implementation of health interventions (Hesse-Biber, 2012).

Contemporary perspectives, such as phenomenology (Husserl, 2012; Merleau-Ponty, 1996) and social constructionism (Berger & Luckmann, 1966; Lock & Strong, 2010), contend that attempting to distill objective data from lived experience is a wrongheaded approach to the study of human behavior. In addition, this narrow focus on specific definitions of rationality limits the questions, theories, and methodologies that can be used by researchers (Susser, 2004). Indeed, numerous writers argue that the study of human beings and their social worlds should not mimic the natural sciences. Instead of separating the world into objective facts and subjective perceptions, all knowledge is understood to involve a knower (Schutz, 1967). In other words, data are always entrenched in some perspective. In this regard, the classic epidemiological triad that seeks to understand health in terms of agent-environment-host demonstrates that the goal should be to try to place disease in an interpretive human context (Page et al., 1995).

## Critical overview

Critics argue that the social and behavioral sciences are more complicated than the natural sciences. Specifically important is that constructs in the social sciences are “second degree,” or models of the concepts that are used by people as they function in their everyday worlds (Schutz, 1953). Because of the theoretical orientations that are dominant in public health, these abstractions are thought to be more valid than the schemas persons use to make sense of their lives. However, this method may obscure how they think about their health or any other issue. Valid concepts, in other words, may be more closely tied to the strategies persons use to interpret and manage events (Berger & Luckmann, 1966).

A development within public health called the “new public health” (Hills, 2000) is an attempt to refocus these efforts, and include community input in evaluations of health and the planning of interventions (Frenk, 1993; Murphy & Rigg, 2014), because the traditional methodologies overlook how people actively construct the realities in which they live. The point is that people do not necessarily think of themselves as composites of risk factors, and quite often decisions and events that are not overtly about health influence how they think about their well-being. In a recent study in the Dominican Republic (Cook, 2013), for example, a direct question about personal health prompted persons to discuss economic and environmental conditions. Most important about this insight is that these persons expect that any effective intervention must extend beyond personal or even interpersonal solutions.

What constitutes a reasonable intervention, therefore, depends on the type of “local reasoning” that is typically not captured by standardized instruments. In this sense, the imagery used in risk factor modeling does not coincide with how people view themselves and their health (Raphael, 2003). As a result, the traditional approach to assessing health has limited the scope of health assessments to variables that can be neatly circumscribed by checklists and introduced into predictive models (Smith, 1998).

For most researchers, only knowledge that is separated from judgments is truthful, and the way to attain this knowledge is by equating reliable and valid data with technical operations (Murphy, 1992). Nonetheless, by seeking to neatly operationalize the factors that persons consider when making decisions regarding their health, researchers can easily lose sight of the context or the web of meaning surrounding health-relevant phenomena. Although there is a place for these types of studies in public health and social science, the first step to any meaningful understanding of health phenomena should not be the obfuscation of the human element.

To be able to understand a phenomenon and create valid constructs that truly represent a group of people, researchers must be comfortable with getting close to the data. Having data that do not fit easily into categorical variables should be valued in research, especially when first investigating a health issue or health behavior in a previously under-studied socioenvironmental context (Quimby, 2006). In order to attain an informed understanding of how people make health-relevant decisions, researchers must move beyond the simple identification of risk factors and grasp how people interpret or construct the meanings of things, events, and relationships. Specifically, health planners should be expected to engage a community in genuine dialog, in order to grasp the various “hidden” issues that might be operating (Murphy & Rigg, 2014; Wallerstein & Duran, 2010).

## Promising strategies underutilized in public health

We suggest three strategies to help accomplish this goal. First, narratives (Agnew, 2006; Charmaz, 1999; Rigg & Murphy, 2013b) are a useful, but underutilized approach that reflects the idea that a person's reality is situational. Illness narratives (Williams, 1984), in particular, provide an innovative and useful way of approaching the subjective aspects of the illness experience (Ezzy, 2000). The norms of a patient's local context, which narratives can help identify, should ideally be used to guide an intervention. The need and course of intervention, in other words, should be driven by the various narratives and storylines in a patient's life and not preconceived disease frameworks (Frank, 1998; Rigg & Murphy, 2013a). Narrative approaches are sensitive to the situations of people (Kleinman & Kleinman, 1996), and can bring into view relevant details of persons’ lives that in most cases are of critical importance to the success of health interventions and programs (Charon, 2011).

Narrative medicine, for example, has been receiving some attention as a new model for clinical practice (Charon, 2001). This approach to medicine recognizes the value of patients’ narratives in practice, research, and education. Proponents of narrative medicine have argued that most medical schools train physicians to treat health problems as simply a medical issue, without taking into account the specific psychological and social history of the patient (Remen, 2002). Narrative medicine places patients’ stories at the center of medical practice and education to encourage a more patient-centered approach to care. Not so much a new specialty as a new frame for clinical work and research, narrative medicine can validate the experience of the patient and encourage physician self-reflection, leading to improved health outcomes (Greenhalgh & Hurwitz, 1999).

Second, photovoice is a research method well-suited to capturing and conveying the point of view of participants. Succinctly defined as a process by which people can identify, represent, and enhance their community through a specific photographic technique (Wang, Cash, & Powers, 2000), photovoice entails providing study participants with cameras, allowing them to record, discuss, and communicate to others their realities as seen through their eyes (Wang & Burris, 1997). The production of a photograph and the photographer's description of the photo provide immediate data and the foundation for building shared knowledge (Newman, 2010). Photovoice is consistent with the core tenets of community-based participatory research (e.g., empowerment, participation) and has been used successfully to address a variety of serious health issues including HIV (Rhodes & Hergenrather, 2007) and chronic pain (Baker & Wang, 2006).

Photovoice methodology has the potential to strengthen the quality and validity of research by using the perspectives of participants to generate new understanding about health-related experiences and decision-making. In this way, the identification and documentation of problems (i.e., barriers to health care utilization) are made by the participants themselves. It is important to note that the use of photovoice also provides an opportunity for vulnerable populations (i.e., homeless) to share their experiences with the research community, service providers, and policymakers; groups that these populations typically have little access to (Lopez, Eng, Robinson, & Wang, 2005). Photovoice methodology sets out to capture and convey the point of view of the participant or patient and allows their experiences to reveal themselves in their own terms (Booth & Booth, 2003). In a recent project (True, 2014), photovoice was used successfully to engage military veterans in communicating their experiences regarding challenges to getting their health care needs met. The data generated by this project not only informed new policy decisions by the Veterans Health Administration but were also used to create a photo exhibit that showed community members how the aftermath of war can affect the health of returning soldiers.

Third, ethnography has the unique capacity to help capture the cultural nuances and context that are often missing from traditional decision-making models. Ethnography is well-suited to resituate and rethink how practitioners conceptualize the health behaviors of persons because of its focus on capturing the social meanings and the ordinary activities of persons in naturally occurring settings (Biehl, 2007). Through the use of participant observation, field notes, and in-depth interviews to capture “everyday life,” ethnographic research reflects the knowledge and the system of meanings in the lives of a particular group. Because of this, ethnography is uniquely qualified to confront and humanize the ways public health problems are framed and interventions carried out (Biehl, 2007).

Although these approaches are not new, they have failed to become mainstream in public health research and are still viewed as ancillary. These approaches have unique potential to bring the experiences of patients into conversations about clinical practice and prevention. Therefore, these and other similar approaches need to become far more normative in public health and epidemiological research.

## “Rational” decision-making

Much of the recent crossover from epidemiological to sociological approaches to public health have focused on creating models that attempt to take into account variables that are beyond the traditional scope of epidemiology. Some of these efforts have focused on neighborhood disorder (Browning & Cagney, 2003; Ross & Mirowsky, 2001), cultural traits (Abraído-Lanza, Chao, & Flórez, 2005; Lee, Sobal, & Frongillo, 2000), inequality (Subramanian & Kawachi, 2003), and the resulting health outcomes. Much of this information, however, is attained through structured questionnaires or analysis of medical records. In general, such studies have the goal of developing path models based on rational choice decisions (Pescosolido, 1992).

Rational choice theory has been adopted by many disciplines (Hechter & Kanazawa, 1997; Li, Zhang, & Sarathy, 2010), and has become widely used to explain a range of behaviors. Rational choice theory focuses on the cost–benefit analyses that actors purportedly make when deciding to take certain actions (Boudon, 1998). Most important at this juncture is that rational choice theory underlies many of the behaviorally based models applied to public health problems, and assumes that people make decisions designed to maximize health outcomes. Recognizing this epistemological influence is incredibly important, especially because research shows that the human mind does not necessarily work “rationally” (Boudon, 1998). That is, persons seem to make relevant decisions that vary along a continuum, instead of pursuing an idealized logic.

The public health literature is replete with models that attempt to describe the decision-making of persons with respect to their health. These models are almost all within the rational choice paradigm and attempt to create formulae designed to predict expected behaviors. But sometimes a person simply does not have the means to act, let alone the luxury to make an informed decision. Examples of commonly used health behavior models in this tradition include the theory of reasoned action-planned behavior model (Montaño & Kasprzyk, 2008; Redding, Rossi, Rossi, Velicer, & Prochaska, 2000), the health belief model (Rosenstock, Strecher, & Becker, 1994; Strecher & Rosenstock, 1997), social cognitive theory (Bandura, 1986, 1990), the transtheoretical model (Prochaska & Velicer, 1997; Redding et al., 2000), and the health behavior model (Andersen, 1995).

These models range from simplistic to complex and include varying levels of contextual variables. The problem is that they all rely on the assumption that human thought and behavior follow a set of predictable rules. What actual community members believe and the value systems that pervade a community is often not the focus of attention. Simply put, the community's world is thus overlooked (Pollner, 2010). Because of this, these types of models, which attempt to map human cognition and decision-making, portray inaccurate representations of how people think and act.

Traditional models in public health approach human decision-making as if people are constantly evaluating objective indices pertaining to health. As researchers work to refine these models—in order to become more holistic—they often seek to identify “missing” variables to understand how to better predict human decisions. But these models assume that the parameters of choosing health are clear, like the rules of a game (Murphy, 1992), and that identical factors influence, although perhaps with different weighting depending on sociodemographic characteristics, the computations of people when they make decisions about their health or seeking treatment.

Consistent with this view of decision-making, behavioral analyses have tended to be equated with risk factor research in public health. Specifically, researchers operationalize variables in order to isolate certain aspects of behavior or the environment (Riffenburgh, 2006). But this approach to modeling health overlooks how persons actively construct the reality in which they live. Comparing studies of condom use based on a rational choice health model versus a more holistic method provides a useful demonstration of the reductionism in these studies.

As HIV becomes an increasingly feminized epidemic (Wingood, 2003), public health researchers try to understand how men and women make safer sex decisions to use protections such as condoms with sexual partners. Attempts to study this issue have traditionally focused on knowledge about disease transmission and condom use. Medical anthropologists, however, have delved into this topic and found that some of the underlying social determinants are a woman's power to insist on condom use (Dunkle et al., 2004; Pulerwitz, Amaro, DeJong, Gortmaker, & Rudd, 2002); how trust, fidelity, and self-esteem are constructed in relationships (Sobo, 1995; Sterk, Klein, & Elifson, 2004; Syvertsen et al., 2013); whether women who are at high risk have alternative economic options aside from receiving financial assistance from their primary partners (Parker, Easton, & Klein, 2000); and the social meaning for women of being perceived as having a faithful partner (Cook, 2013; Sobo, 1995). These types of in-depth studies that look at the social meaning of condom use can more accurately assess the situation from the perspective of the actors, and therefore lead to more socially sensitive and, thus, efficacious interventions.

## Conclusion

As a discipline, public health is struggling to advance the third wave that moves beyond individual level behaviors to critically understand health in the community context. This transition includes more holistic definitions of health and broadens what is thought to fall under the purview of health, including a critique of the dualistic assumptions that underpin public health and health care. Understanding the assumptions of traditional public health is important to appreciate their impact on health-related decision-making.

The connection between theory and public health is important, particularly in view of recent theoretical shifts that define knowledge and alter how decision-making is evaluated (Ferguson, 2006). This manuscript provides a critique of traditional public health approaches and theoretically justifies what and why changes are warranted. The overarching point is that the process of overanalyzing variables is impeding the process of gaining insight into the experiential-based pathways persons follow when defining health and seeking treatment. This claim is especially important to behavior change and health decision-making, because intuitive and subtle interpretations influence the decisions that people make (Krieger, 2005). Strategies that are grounded in the traditions of, for example, phenomenology or participatory action research, can be used to understand not just knowledge about disease transmission, but how people view their options, barriers, and the likely success of a particular intervention.
